# A liquid metal-embedded 3D interconnected-porous TPU/MXene composite with improved capacitive sensitivity and pressure detection range†

**DOI:** 10.1039/d4ra01215a

**Published:** 2024-05-14

**Authors:** Zhong Zheng, Xing Fang, Yifan Pan, Shuyi Song, Huan Xue, Jun Li, Yi Li, Jing Li

**Affiliations:** a Hubei Key Laboratory of Modern Manufacturing Quantity Engineering, School of Mechanical Engineering, Hubei University of Technology Wuhan Hubei 430068 China lijing@hbut.edu.cn; b Renal Division and Division of Engineering in Medicine, Department of Medicine, Brigham and Women's Hospital, Harvard Medical School Boston MA 02115 USA

## Abstract

Flexible capacitive sensors are widely deployed in wearable smart electronics. Substantial studies have been devoted to constructing characteristic material architectures to improve their electromechanical sensing performance by facilitating the change of the electrode layer spacing. However, the air gaps introduced by the designed material architectures are easily squeezed when subjected to high-pressure loads, resulting in a limited increase in sensitivity over a wide range. To overcome this limitation, in this work, we embed the liquid metal (LM) in the internally interconnected porous structure of a flexible composite foam to fabricate a flexible and high-performance capacitive sensor. Different from the conventional conductive elements filled composite, the incompressible feature of the embedded fluidic LM leads to significantly improved mechanical stability of the composite foam to withstand high pressure loadings, resulting in a wider pressure sensing range from 10 Pa to 260 kPa for our capacitive composite sensor. Simultaneously, the metal conductivity and liquid ductility of the embedded LM endow the as-fabricated capacitive sensor with outstanding mechanical flexibility and pressure sensitivity (up to 1.91 kPa^−1^). Meanwhile, the LM-embedded interconnected-porous thermoplastic polyurethane/MXene composite sensor also shows excellent reliability over 4000 long-period load cycles, and the response times are merely 60 ms and 110 ms for the loading and unloading processes, respectively. To highlight their advantages in various applications, the as-proposed capacitive sensors are demonstrated to detect human movements and monitor biophysical heart-rate signals. It is believed that our finding could extend the material framework of flexible capacitive sensors and offer new possibilities and solutions in the development of the next-generation wearable electronics.

## Abstract 2

We embed the LM in the internally interconnected porous structure of flexible foam, in which the LM endows the as-fabricated capacitive sensor with outstanding pressure sensitivity.

## Graphical abstract

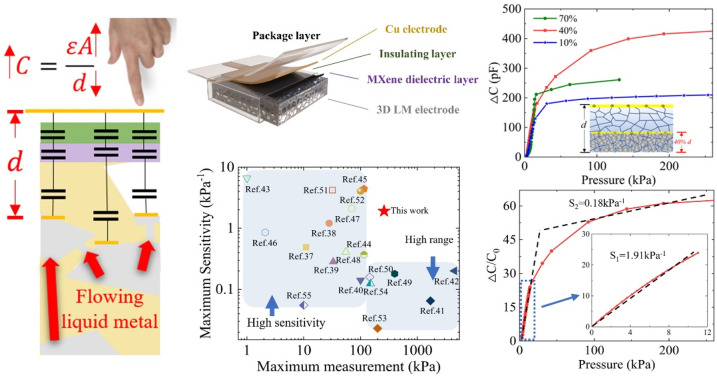

## 1. Introduction

With the growing interest in future portable electronics, flexible electromechanical sensors, which can convert the environmental physical stimuli into measurable electrical signals,^1^ have attracted substantial research attention. Their applications in wearable electronic devices include health monitoring,^2–4^ human–machine interaction,^5,6^ and the Internet of Things.^7–9^ The electromechanical sensors are usually constructed based on the electromechanical sensing mechanisms of piezoresistive,^10^ capacitive,^11^ piezoelectric,^12^ and triboelectric.^13^ Among them, the capacitive sensor has emerged as a favorable option due to its low hysteresis, low power consumption, and suitability for extensive application in wearable devices.^14^

Capacitive sensors also have the advantage of a simple structure. Capacitive pressure sensors usually take the form of parallel plate capacitors, which consist mainly of two parts: the parallel electrode layer and the dielectric layer sandwiched between the electrode layers.^15^ The capacitance value of the capacitive pressure sensor is determined by the electrode layer spacing, the overlapped area of electrodes, and the dielectric constant of the dielectric layer.^16^ In order to improve their sensing performance many researchers have been devoted to the improvement of their sensing performance by facilitating the change of the electrode layer spacing.^17^ The deformability of the electrode layer spacing depends on the modulus of elasticity of the material used for the sensor. Design solutions such as cylinders,^18^ pyramids,^19^ microspheres,^20^ and micro/nanostructures of plant templates^21,22^ have been used. These studies enable the sensors to obtain a larger longitudinal deformation space by generating an air gap, which facilitates the change of electrode layer spacing during compression.^23^ In addition, during compression, the air gap is replaced by the surrounding material, resulting in an increasing dielectric constant.^24^ However, when subjected to high-pressure loads, the air gaps introduced by these structures are easily squeezed, resulting in a limited increase in sensitivity over a wide range.^25^

Unlike capacitive sensors based on solid materials, gallium-based liquid metals (LMs, *e.g.*, eutectic gallium indium, EGaIn), which are essentially liquid, have better deformability and offer a new option for capacitive sensor design.^26,27^ In particular, EGaln, which consists of 75.5 wt% gallium and 24.5 wt% indium, is widely recognized as one of the liquid metal alloys suitable for use in the field of flexible electronics due to its excellent fluidity,^28^ nontoxicity,^29–31^ and high electrical conductivity (3.4 × 10^4^ S m^−1^).^32^ A number of flexible capacitive pressure sensors based on liquid metals have been designed and prepared. For example, Zhang *et al.* prepared capacitive pressure sensors based on icicle-shaped liquid metal film electrodes by injecting EGaIn into Ecoflex elastomer channels, the sensor has been demonstrated with high sensitivity of 0.39 kPa^−1^ in the range of 0–1 kPa.^33^ The feasibility of the liquid metal material as the electrode of capacitive sensors was demonstrated. On the other hand, Yang *et al.* improved the sensing performance of the sensor by mixing EGaIn particles with elastomers as a dielectric layer for capacitive sensors, and prepared a soft capacitive pressure sensor based on a two-layer liquid metal elastomer foam with a sensitivity as high as 0.073 kPa^−1^ and a sensing range as high as 280 kPa.^34^ Zhang *et al.* reported a capacitive sensor by covering the surface of PDMS with EGaIn as an electrode and used a femtosecond laser to prepare a capacitive sensor with a super-metallic hydrophobic double-sided polydimethylsiloxane micro-pyramid array as the dielectric layer. With a sensitivity of up to 2.78 kPa^−1^ but a sensing range of only 20 kPa, the trade-off between high sensitivity and wide pressure range needs to be overcome, although the improved sensing performance of these designs is impressive.^35^ Also, some of the high-precision structure preparation processes greatly increase the production cost of the sensors, and the preparation process is time-consuming. Therefore, there is an urgent need for a low-cost and simple strategy to fabricate capacitive sensors with high sensitivity over a wide sensing range.

In this work, we are inspired by the fact that the liquid metal (LM) has unique characteristics of metal conductivity, liquid ductility, and low volume viscosity that allow them to flow and maintain high conductivity after compression when compared with the solid metal electrode layer. At the same time, the liquid metal can be filled into the polymer as a conductive filler to prepare a flexible polymer-LM filler. The filled liquid phase can be easily deformed to match the deformation of the matrix. This characteristic fits well with the demand for capacitance sensors for changing the electrode layer spacing and provides a new design idea for improving sensitivity. Therefore, we constructed an easy-to-fabricate TPU/MXene/LM composite capacitive sensor (TMLS) that requires no complex instrumentation and special preparation conditions during the preparation process. As shown in Fig. S1a,† it utilizing the MXene free-standing film as the dielectric layer and the LM embedded in the thermoplastic polyurethane (TPU) skeleton is used as the conductive electrode. However, the original TPU skeleton has poor wettability with liquid metal, and liquid metal cannot be stably embedded and adhered. The purpose of using polymethacrylate (PMA) to modify the TPU skeleton is to fix the EGaIn liquid metal in the TPU skeleton and to form a stable liquid metal electrode layer. When the external pressure leads to the compression of the TPU skeleton, the liquid metal flows dynamically in the interlinked pores of the TPU foam. The compressive flow of the liquid metal can lead to a greater change in the electrode layer spacing. Many micro-capacitors composed of liquid metals with different heights in each hole integrate a more sensitive total capacitance response, as shown in Fig. S1b.† Based on this principle, the liquid metal layer in the compressible TPU skeleton is used as the pressure sensitive layer. The liquid metal is distributed in three-dimensional space, and the distance from the upper electrode is a gradient, which contributes to the continuous change of the capacitance value of the sensor to meet the pressure load detection. As a dielectric layer material for flexible capacitive sensors, MXene (Ti_3_C_2_T_X_) is highly competitive due to its unique two-dimensional layered structure, high dielectric constant, good mechanical properties (high flexibility and high strength) and large specific surface area. Therefore, the MXene (Ti_3_C_2_T_X_) film replaces the elastic dielectric layer commonly used in the current flexible capacitive pressure sensor. The self-supporting MXene film dielectric layer no longer bears the compressible function and does not need to reserve excess deformation space. This ultra-thin dielectric layer can bring a higher basic capacitance value (6.8 pF) so that the sensor can obtain a higher signal-to-noise ratio. Experimentally, the as-fabricated capacitive sensor displays a high sensitivity of 1.91 kPa^−1^ within the pressure range of 10 Pa–25 kPa and concurrently maintains a good sensitivity of 0.18 kPa^−1^ up to 260 kPa pressure loading. As a comparison, the comprehensive performance of the as-fabricated capacitive sensor is superior to other capacitive pressure sensors based on a porous dielectric layer or porous electrode. Moreover, our sensor exhibits favorable stability and prolonged durability and no occurrence of the signal damping phenomenon during over 4000 cycles of under-pressure loading-unloading. As a conceptual validation, we developed a prototype to elucidate the potential applications of TMLS in the realms of human machine interaction and smart wearable devices.

## 2. Experimental methods

### 2.1 Materials

EGaIn liquid metal (≥99.99%), which is composed of 75.5 wt% gallium and 24.5 wt% indium, is from Hunan Zhongcai Shengte New Material Co., Ltd. Layered ternary carbide Ti_3_AlC_2_ powder (MAX phase, 200 mesh) was provided by Jilin eleven technology company. 3M Tegaderm from 3M Medical Devices Co., Ltd. Polyimide (PI) tape from Shanghai Mingshen Electronic Technology Development Co., Ltd. Thermoplastic polyurethane (TPU) comes from BASF, Germany. Cubic single-crystal sucrose particles are from Domino Foods and are used as sacrificial templates. Lithium fluoride (LiF, AR, 98%), concentrated hydrochloric acid (36–38%), anhydrous ethanol, *N*,*N*-dimethylformamide (DMF) and tetrahydrofuran (THF) were provided by Sinopharm Chemical Reagent Co., Ltd. Deionized water was used in all the experiments.

### 2.2 Preparation of 3D interconnected liquid metal electrode layer

#### Preparation of foam skeleton

**Fig. 1:**
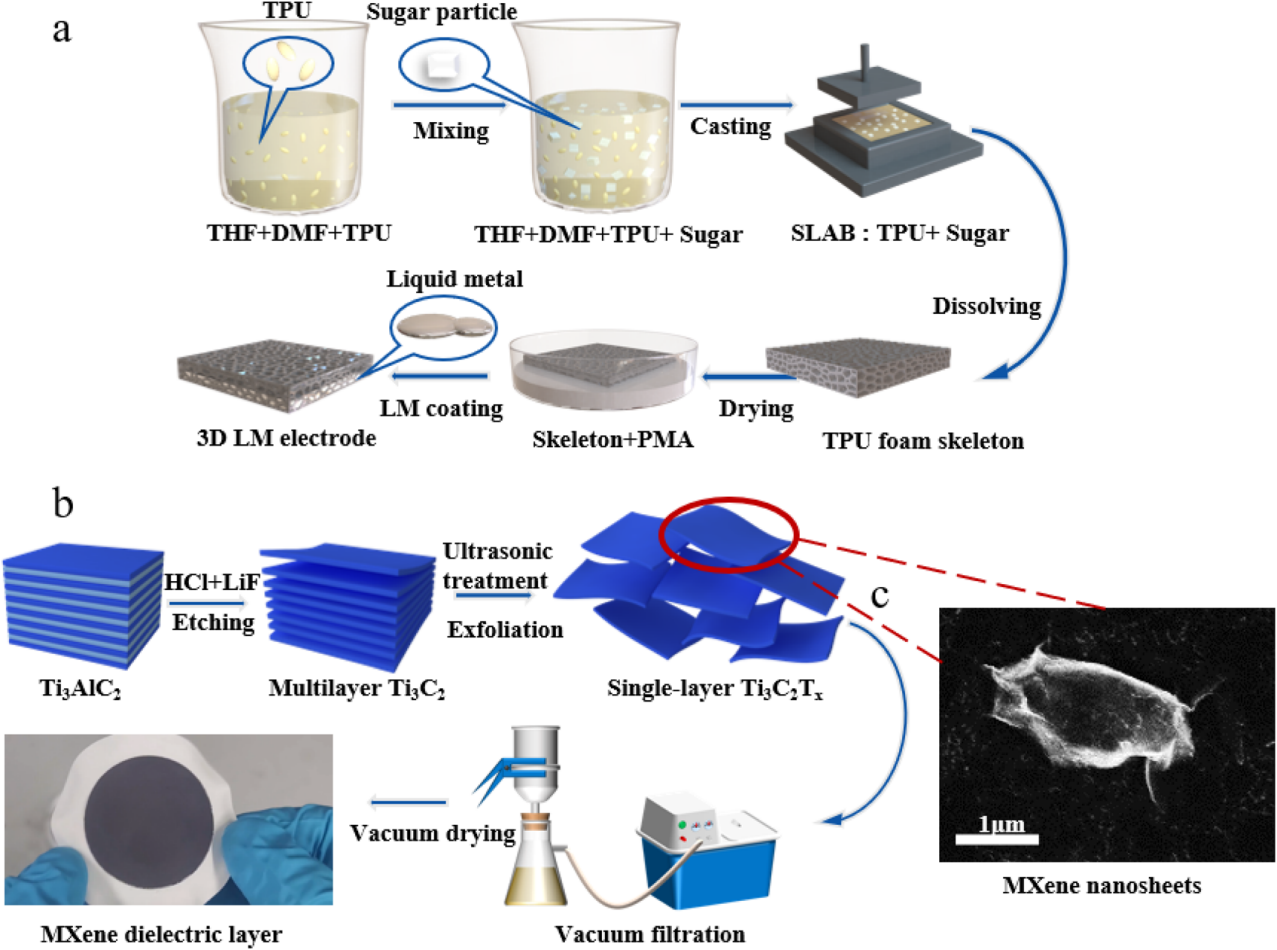
The fabrication process of the as-proposed TMLS (a) fabrication process of the TPU foam and (b) MXene free-standing film. (c) SEM image of prepared MXene (Ti_3_C_2_T_X_) nanosheets.

TPU foam skeleton with elasticity and high flexibility was prepared by sacrificial template method. As shown in Fig. 1a, in order to prepare high concentration TPU solution by dissolving solid TPU particles, a mixed solvent of DMF and THF (volume ratio 1 : 1) was configured. The solid TPU particles were added to the prepared DMF and THF mixed solvent and heated and stirred for 5 h at 50 °C and 100 rpm using a magnetic heating plate stirrer to obtain a uniform TPU mixed solution. Cubic single crystal sucrose particles were filtered through a metal mesh with a pore size of 1 mm to remove sugar particles larger than 1 mm. Sugar particles were added to the TPU mixed solution and fully stirred. The mixed solution was cast in a designed mold and die-cast at 80 °C for 12 hours. In the environment of 80 °C, DMF and THF solution will quickly volatilize to form a mixed block of TPU and sugar particles. Then, the sugar particles were dissolved in deionized water to obtain TPU porous structure foam. The TPU foam was cut into thin sheets with a specific thickness (5 mm) by a die-cutting machine, and the impurities were removed by washing with 200 mL deionized water and ethanol to obtain a TPU porous foam skeleton.

#### Foam skeleton modification

The unmodified TPU skeleton has poor wettability with liquid metal and cannot adhere stably. The effect of polymethacrylate modification is to fix EGaIn liquid metal in the TPU skeleton to form a stable liquid metal electrode layer. The specific operation is to increase the fluidity of the polymethacrylate polymerization emulsion by adding 25 wt% deionized water to the polymethacrylate emulsion and fully stirring. Then, the bottom of the TPU foam skeleton slice obtained in the previous step was immersed in the above solution and impregnated for 1 h at room temperature (about 20 °C). Polymethacrylate-modified TPU foam skeleton was obtained by drying at 60 °C for 2 hours.

#### Preparation of electrode layer

The polymethacrylate modified TPU foam skeleton obtained in the previous step was partially immersed in a liquid metal pool, so that the liquid metal was embedded in the interconnected pores of the modified TPU foam and adsorbed on the polymethacrylate modified pore wall to obtain a liquid metal layer.

### 2.3 Preparation of MXene dielectric layer

A 4.5 g lithium fluoride (LiF, Sinopharm, >98%) was added to 60 mL hydrochloric acid (HCl, Sinopharm, 9 M) in a plastic beaker (100 mL) and stirred for 5 min to obtain an etching solution. Then, 3 g of Ti_3_AlC_2_ was slowly added to the solution and stirred at 40 °C for 24 hours. The obtained suspension was centrifuged at 6000 rpm and washed with deionized water until the pH of the supernatant exceeded 6 to obtain MXene (Ti_3_C_2_T_X_) nanosheets. High-quality MXene (Ti_3_C_2_T_X_) nanosheets exhibit a clear sheet structure with a lateral length of 1–2 μm, as shown in the high-resolution SEM image of Fig. 1c. The suspension of these MXene remained uniform after 24 h, indicating that MXene can be stably dispersed in aqueous solution.

Next, the above MXene nanosheets were re-dispersed in 100 mL deionized water and ultrasonically treated in ice water for 1 hour. Vacuum-assisted filtration was used to deposit MXene on an aqueous microporous filter membrane with a pore size of 0.22 μm, as shown in Fig. 1b. Then, it was vacuum dried at 60 °C for 24 h to obtain an MXene free-standing film with a thickness of about 100 μm and cut into the required size.

### 2.4 Assembly of TMLS

In order to construct TMLS, upper flexible copper film electrode, MXene free-standing film, and TPU foam skeleton/3D liquid metal was cut into the same length and width shape. As shown in Fig. S1a,† the dielectric layer is an MXene free-standing film with a thickness of only 100 nm, which is placed on the upper layer of the TPU foam skeleton. The electrode layer and the pressure sensitive layer are TPU foam skeleton/3D liquid metal layer. The insulating layer is a transparent and soft 3M Tegaderm, which is employed to prevent short circuits and block liquid metal leakage. Finally, two transparent and soft 500 nm ultra-thin PI tapes were used to encapsulate the above multilayers. The purpose of the package is to fix the relative position of the upper and lower electrodes and other layers while isolating the external environment signal interference.

In addition, to compare and verify, according to the structural characteristics of the most typical flexible capacitive pressure sensor, we prepared three flexible capacitive pressure sensors: porous TPU sensor (TS), porous TPU/MXene sensor (TMS), and porous TPU/LM sensor (TLS). All porous TPUs were prepared from the same batch of sugar templates by the same sacrificial template method in Section 2.2 of this article. ① Porous TPU sensor: the prepared TPU foam skeleton was used as a dielectric layer. The dielectric layer is sandwiched between two flexible copper film electrodes. ② Porous TPU/MXene sensor: a layer of MXene free-standing film (dielectric layer) was added to the upper surface of the prepared TPU foam skeleton. MXene free-standing film is conductive. Therefore, in order to prevent the short circuit phenomenon, an insulating layer (3M Tegaderm) is added between MXene free-standing film and the flexible copper film electrode layer to make the entire pressure sensor capacitive. ③ The porous TPU/LM sensor: the electrode layer embedded with liquid metal in the TPU porous interconnected foam skeleton was prepared by the same method and process in Section 2.2. After the flexible copper film electrode is pasted on its upper surface, it is directly packaged with a 3M Tegaderm insulation layer.

### 2.5 Characterization and measurement

The structure of the dielectric layer was examined by scanning electron microscopy (SEM, Mira, Tescan, Czech Republic). The most important components in the sensor performance test are pressure application and signal acquisition equipment. The dynamic pressure is controlled and measured by the universal testing machine (CMT4204, Meters Industrial Systems Co., Ltd., China), and the corresponding capacitance change is measured by the LCR meter (TH2832, Tonghui Co., Ltd., China). The sensor sample is fixed on the sample platform of the universal testing machine and connected to the LCR meter using wires. Place the computer to set the pressure and record the change in the capacitance value.

#### Arterial pulse measurement

Arterial pulse was measured in a passive state. In order to detect wrist radial artery pulsation. Experiments involving human subjects: experiments involving human subjects were performed with the full informed consent of the volunteers. All reported tests meet the ethical requirements of Hubei University of Technology.

## 3. Results and discussion

### 3.1 TPU foam characterizations

**Fig. 2:**
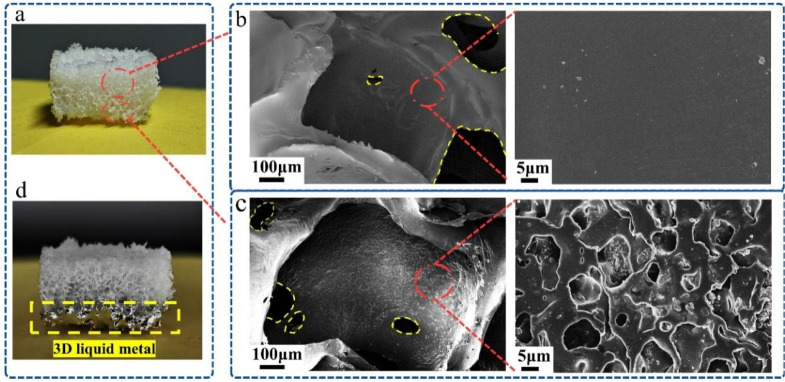
Characterization of the as-fabricated liquid metal-embedded TPU/MXene composite. (a) Photos of modified TPU foam (upper half unmodified, lower half modified). SEM image of (b) a cell of unmodified TPU foam and its inner wall, (c) a cell of modified TPU foam and its inner wall. (d) Photos of TPU foam embedded in liquid metal.

The digital photos (Fig. 2a) and SEM images (Fig. 2b and c) of the TPU foam before and after polymethacrylate modification indicate that cube cavities are densely distributed in the white TPU foam, and most of them have a side length of about 600 μm. These cubes are interconnected, forming a network of controllable pores with irregular gaps ranging from 100 μm to 300 μm in width. This demonstrates that we can obtain controllable and interconnected pores with controllable shapes and sizes by using sieved uniform sugar particles as sacrificial templates to prepare TPU foam skeleton, ensuring its reproducibility, which facilitates large-scale repeated preparation of porous interconnected-porous skeleton. Moreover, the TPU foam skeleton we prepared can be easily processed into various thicknesses and shapes according to flexible sensors' requirements, demonstrating its versatility. These interconnected pores are mainly employed to generate air gaps and accommodate liquid metals, ensuring the smooth flow of liquid metals within the pores to establish a continuous current path network.

The inner walls of the cavities and pores in the TPU foam (Fig. 2b) before polymethacrylate modification were smooth. The high surface energy of the liquid metal produces a strong surface tension. The poor wettability between the liquid metal and the smooth TPU inner wall before polymethacrylate modification, coupled with its extremely high fluidity, makes it difficult to embed and adhere to the TPU foam skeleton. Following polymethacrylate modification, the inner walls (Fig. 2c) underwent a transformation, becoming rough and featuring numerous micro- and nano-sized pores. The wettability of these inner walls with the liquid metal surface significantly improved, facilitating successful embedding and adherence.

**Fig. 3:**
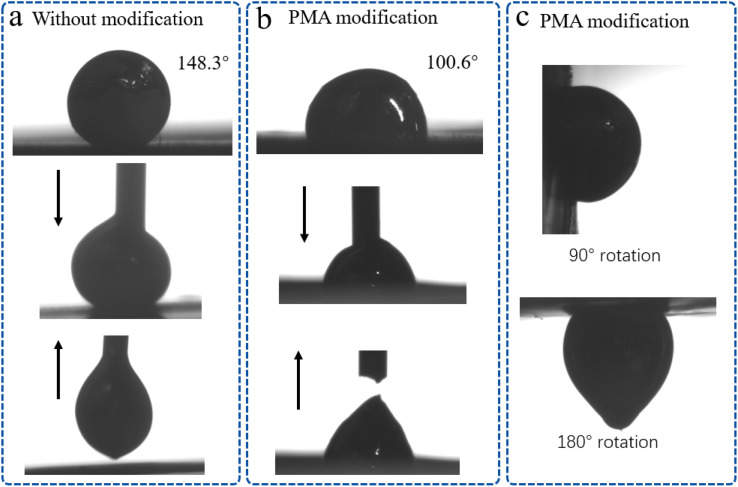
The adhesion test of EGaIn on the surface of TPU before and after modification. (a) The contact angle between EGaIn and unmodified TPU surface and the image in the lifting test. (b) The contact angle of EGaIn and PMA modified TPU surface and the image in the lifting test. (c) Images of liquid metal droplets rotating 90° and 180° after contacting PMA modified TPU surface.

In order to show the wettability change of liquid metal more intuitively, the adhesion test of liquid metal before and after TPU modification was also carried out. As shown in Fig. 3a and b, the contact angles of unmodified TPU and PMA-modified TPU with liquid metal droplets were 148.3° and 100.6°, respectively. In addition, the lifting experiment revealed that the liquid metal can be effortlessly removed from the unmodified TPU surface without leaving any residue. However, the liquid metal droplet contact modified TPU surface proved to be more challenging to lift. Finally, as illustrated in Fig. 3c, following contact between the liquid metal droplets and the PMA-modified TPU surface, the droplets can be fixed in place, even when the overall rotation is 90° or 180°. This demonstrates that EGaIn has excellent wettability with the modified TPU skeleton. Following the PMA modification, it can be guaranteed that the movement of the liquid metal within the TPU skeleton is a consequence of the deformation of the skeleton caused by stress and that it will not flow around due to its own gravity and other factors. This enhancement can be attributed to the aliphatic groups of acrylic acid formed on the inner wall's surface through polymethacrylate modification. These groups can establish hydrogen bonds with the oxide layer of the liquid metal,^36^ serving as anchor points. The schematic diagrams in Fig. S2† show the chemical interaction between PMA and EGaIn. The FTIR spectrum is shown in the figure. For TPU, 2943 cm^−1^ and 2858 cm^−1^ are the stretching vibration absorption peaks of C–H. The stretching vibration absorption peak of C=O in carbamate on TPU is at 1701 cm^−1^, and the bending vibration absorption peak of N–H is at 1525 cm^−1^, which is the characteristic absorption peak of TPU. The stretching vibration absorption peak of C=O in carbamate on TPU/PMA is at 1728 cm^−1^. The introduction of PMA leads to the formation of hydrogen bonds between C=O bonds and N–H bonds in other segments, resulting in the shift of group peaks. The curve in Fig. S3† shows an absorption peak shift of the C–H bond. These results suggested that there may be hydrogen-bond interaction between the Ga_2_O_3_ and the hydrogen atom of the methyl. As shown in Fig. 2d the liquid metal can adhere well to the surface of the TPU foam skeleton, which strongly proves the importance of polymethacrylate.

The significant air gap created by the porous structure in both the TPU foam skeleton and the flexible TPU substrate significantly enhances the compressibility of the TPU foam. This facilitates the generation of deformations, including compression, bending, and twisting. As a result, it can also function as a pressure-sensitive element in the flexible capacitive pressure sensor. Manual compression can easily reduce it by more than 70%. When the porous TPU skeleton is compressed, the foam skeleton buckles and collapses, and the air gap is eliminated by extrusion. This results in a lower initial compression modulus and allows a greater compression strain compared to the bulk TPU under the identical condition, this gives the flexible sensor higher sensitivity. Simultaneously, our prepared foam skeleton effortlessly returns to its initial size after the release of pressure.

### 3.2 Pressure sensing performance

#### 3.2.1 Capacitance response and sensitivity

**Fig. 4:**
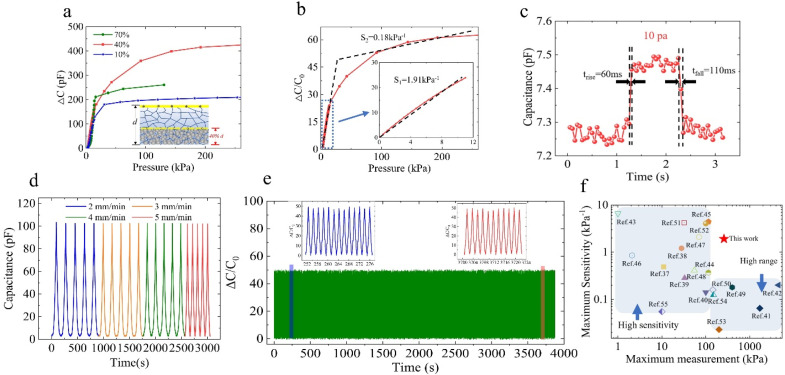
Electromechanical response of the as-fabricated capacitive pressure sensors. Pressure–capacitance variation curve of (a) TMLS when the volume ratio of liquid metal in TPU foam is 10%, 40%, 70%, (b) TMLS containing 40% liquid metal (after calculating the normalized). (c) Response and recovery time test of TMLS under a pressure load of 10 Pa. (d) Capacitance response curves during cyclic compression at different frequencies. (e) Working stability test of TMLS under repeated pressure loading (50% deformation)–unloading for 4000 cycles. (f) Comparison of TMLS with state-of-art counterparts.

Here, we define the response value of the sensor as the variation of the capacitance value (Δ*C*) after the load pressure on the sensor surface, where Δ*C* = *C*–*C*_0_. When the volume ratio of liquid metal in TPU skeleton is 10%, 40%, and 70%, respectively, the pressure–capacitance change response curve (kPa–Δ*C*) of our TMLS to load pressure is shown in Fig. 4a. As the load pressure increases, it is evident that the capacitance response values of TMLS with three liquid metal contents all indicate a steady upward trend, displaying a proportional relationship with the applied pressure. Among them, the capacitance response of TMLS containing 40% liquid metal demonstrates the most favorable capacitance response, characterized by a stable, continuous, and gradual increase in the curve, significantly outperforming TMLS with other liquid metal contents. In addition, the TMLS containing 70% liquid metal has the fastest capacitance response (sharp rise in the curve) in the small load pressure range. However, when the pressure load exceeds 120 kPa, it fails prematurely. This underscores the contribution of the liquid metal and air gap within the TPU skeleton to the capacitance response of TMLS, and the final capacitance response value of TMLS containing 40% liquid metal is twice as high as that of TMLS containing 10% liquid metal, which also shows that the contribution of liquid metal to capacitance response is very large. The related mechanism will be analyzed and discussed in Section 3.4 of this paper.

Here, the sensitivity of the sensor is *S* = (Δ*C*/*C*_0_)/Δ*P*, where Δ*C* is the capacitance response value, *C*_0_ is the initial capacitance, and Δ*P* is the change pressure. Due to the large initial resistance, after calculating the normalized, the use of (Δ*C*/*C*_0_) as the calculation element of sensitivity will artificially underestimate the performance of TMLS. However, the capacitance response curve (Fig. 4b) of TMLS containing 40% liquid metal demonstrate that it has a stable capacitance response both under small pressure (<10 kPa) and high pressure load (<260 kPa). The response curve has obvious linear characteristics in the small pressure range.

To further compare and verify the sensing performance of our structurally innovative flexible capacitive pressure sensor, we adopted the same batch of sugar templates to prepare four flexible capacitive pressure sensors by sacrificial template method: porous TPU sensor (TS), porous TPU/MXene sensor (TMS), multi-TPU/LM sensor (TLS) and our TMLS, as shown in Fig. S4a.† The first three structures well represent the latest research progress of flexible capacitive pressure sensors. Measurement results in Fig. S4b† indicate that the four flexible sensors can maintain the stable response of the capacitance value and the response value is proportional to the pressure during the continuous pressure loading. This is attributed to the excellent mechanical properties and structural stability of TPU porous foam as a pressure-sensitive layer, as mentioned above. The capacitive response value of the TMLS is much higher than that of the other three pressure sensors under the same compressive load. Impressively, it is more than 3300% higher than that of the porous TPU sensor (from 12.5 pF to 425.2 pF), and more than 460% higher than that of the porous TPU/MXene sensor.

Here, our calculation results (Fig. S4c†) clearly demonstrate that the sensitivity of TMLS is higher than that of the other three pressure sensors, whether under low pressure loading (<10 kPa) or high pressure loading. It can also be seen that although the final capacitive response value TMS is higher, thanks to the addition of the liquid metal, TLS obtains better sensitivity at high pressure. The incompressible nature of the embedded fluid LM leads to a significant improvement in the mechanical stability of the composite foam when subjected to high-pressure loads, and the pores are not easily and easily crushed, thus providing the sensor with a wider pressure sensing range and sensitivity. Moreover, the initial capacitance signal of TMLS is increased by more than 300% (from 2.0 pF to 6.8 pF) compared to the other three pressure sensors. This also means that TMLS has a higher SNR.

#### 3.2.2 Other sensing properties

For the purpose of detecting the stability of this structurally innovative capacitive pressure sensor in practical application scenarios, 4 kPa pressure signals with different frequencies ranging from 2–5 mm s^−1^ were applied to the sensor surface. The curve in Fig. 4d shows that TMLS can consistently deliver a stable response under different frequencies of external pressure stimulation. Moreover, for the benefit of testing the pressure minimum detection limit and response time of TMLS, we rapidly placed TPU particles (10 Pa) on TMLS and removed them after about 1–2 seconds. The capacitance response curve (Fig. 4c) manifests a significant step, and the response signal is clearly distinguishable from the noise signal. This demonstrates that its minimum pressure detection limit can reach 10 Pa, indicating the ability of TMLS to detect subtle external pressure. Meanwhile, TMLS can respond swiftly within 60 ms, and its initial capacitance value increases rapidly to a stable state after the pressure loading. Its initial capacitance value quickly returns to the initial value within 110 ms after unloading. For evaluating the test and the long-term reliability of the sensor performance, a pressure loading–unloading cycle of 50% deformation was carried out for 4000 cycles. According to the capacitance response output curve (Fig. 4e), the capacitance response signal under long-term pressure load is stable, continuous, and reproducible, and there is no signal damping phenomenon, which demonstrates that TMLS has excellent robustness. Whether in the low-pressure detection range (<25 kPa) or in the higher range (25 kPa to 260 kPa), the sensitivity of TMLS with innovative structure is better than that of other capacitive pressure sensors based on porous dielectric layer or porous electrode layer reported in the past five years, as shown in Fig. 4f.^37–55^

### 3.3 Mechanism analysis

For the benefit of better understand the sensing mechanism of this structurally innovative capacitive pressure sensor, we use a simplified equivalent circuit to establish a model of TMLS and analyze the influence of the mechanical deformation behavior on the sensor's capacitive response output.

TMLS can be regarded as a parallel plate capacitor, dividing the area of the sensor by *n* blocks, so the equivalent circuit of the flexible sensor can be described by *n* columns.1
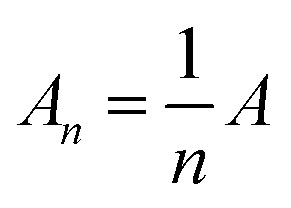


The series part of each block can be calculated by the following series capacitance calculation formula.2
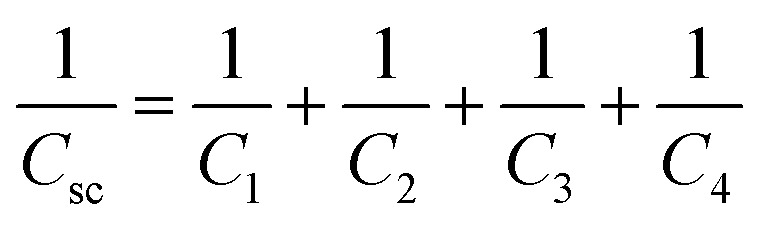
3
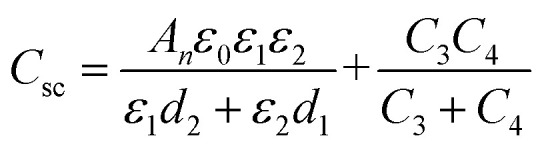


In formula (2), *C*_1_ and *C*_2_ are capacitors based on the air layer and TPU skeleton, respectively. *C*_3_ and *C*_4_ are capacitors based on Tegaderm encapsulation layer and MXene dielectric layer. *C*_sc_ is the equivalent capacitance of the series capacitor. Formula (3) gives the detailed calculation method of *C*_sc_.

Where *ε*_0_ is the vacuum dielectric constant, *ε*_1_ and *ε*_2_ are the relative dielectric constants of air and TPU skeleton. The thickness of the air layer and the TPU are denoted as *d*_1_ and *d*_2_, respectively, and the values of *d*_1_ and *d*_2_ decrease when the pressure load is applied.

TMLS has a higher SNR than the other three pressure sensors. This can be attributed to several factors. Firstly, TMLS uses MXene free-standing film as the dielectric layer. The adjacent MXene nanosheets in the dielectric layer are stacked, form numerous interlayers micro capacitors. Leveraging the high dielectric constant of MXene itself, the sensor achieves a higher dielectric constant and initial capacitance value. When subjected to the same pressure load, although there is not much difference in the compressive strain generated by the four flexible sensors under the same pressure loading, MXene free-standing film dielectric layer imparts a higher dielectric constant to TMLS, thereby significantly improving its sensitivity. Different from the elastic dielectric layer used in other flexible capacitive pressure sensors, we integrate the 3D dynamic liquid metal electrode layer into the compressible TPU skeleton, simultaneously serving as a pressure-sensitive layer. MXene free-standing film with a thickness of only about 100 μm is employed as the dielectric layer to make it no longer bear the compressible function. MXene free-standing film and Tegaderm can maintain their shape stability under pressure loading, and will not interfere with the electrical properties of TMLS due to the thickness deformation.

The high compliance of 3D interconnected liquid metal and TPU foam skeleton is the key to improving the sensitivity of TMLS. While applying pressure load, it's essential to highlight that the liquid metal in the electrode layer is distributed in three-dimensional space. The distance from the upper electrode changes gradually, facilitating a continuous alteration of the sensor's capacitance value to accommodate pressure load detection at various levels. Since the pressure sensitive layer of the four flexible sensors is based on TPU porous foam, the air gap in the TPU foam skeleton is continuously squeezed and eliminated. Is continuously eliminated during the same continuous loading pressure, and the distance between the plates of the capacitor changes. The four flexible sensors have a stable response to the capacitance value. For TMLS, in addition to air gap extrusion, several factors contribute to its high sensitivity. First, when the pressure is loaded, the distance between the plates of the capacitor undergoes changes due to the variations in the thickness *d*_1_ + *d*_2_ of the air layer and the TPU. Secondly, the TPU skeleton produces mechanical deformation, the contact area of the inner hole wall also changes, the air is squeezed out of the air gap decreases, and the dielectric constant *ε*_1_ changes compulsorily. Thirdly, in the initial phase of the process within TMLS, the 3D liquid metal electrode layer and the dielectric layer are separated by the TPU skeleton and the air gap, constituting a non-contact area. With the rise in pressure load, the TPU foam chamber containing the liquid metal experiences compression and deformation. This compels the liquid metal to flow into other interconnected pores and the surrounding area of the compressed site, leading to a subsequent reduction in the relative spacing between the flexible capacitor plates. Fourth, in the later stage of compression, as the pressure further increases, the liquid metal begins to squeeze the tape encapsulation layer, resulting in lateral flow, which increases the area *A*_*n*_ of the liquid metal electrode layer. Until the porous structure of the TPU skeleton is compressed to the limit, the bottom of the 3D liquid metal electrode is fully expanded, and the maximum capacitance value is obtained. This makes TMLS have high sensitivity under high pressure load.

Given the analysis of the mechanism above, one can comprehend the capacitive response performance of TMLS with three different liquid metal contents, as illustrated in Fig. 4a. It is evident that TMLS with a moderate liquid metal content, such as 40%, effectively utilizes the functions of liquid metal and air gap. With an excessive liquid metal content, for instance, 70%, the packaging layer is unable to adequately contain the surplus liquid metal under substantial load pressure. This leads to leakage, causing the sensor to fail as a whole and limiting the sensing range. In instances where the liquid metal content is too low, for instance, 10%, the air gap in the TPU foam is already expelled under load pressure. The subsequent impact of the liquid metal on the capacitance is constrained, resulting in the lowest final capacitance response value and sensitivity.

**Fig. 5:**
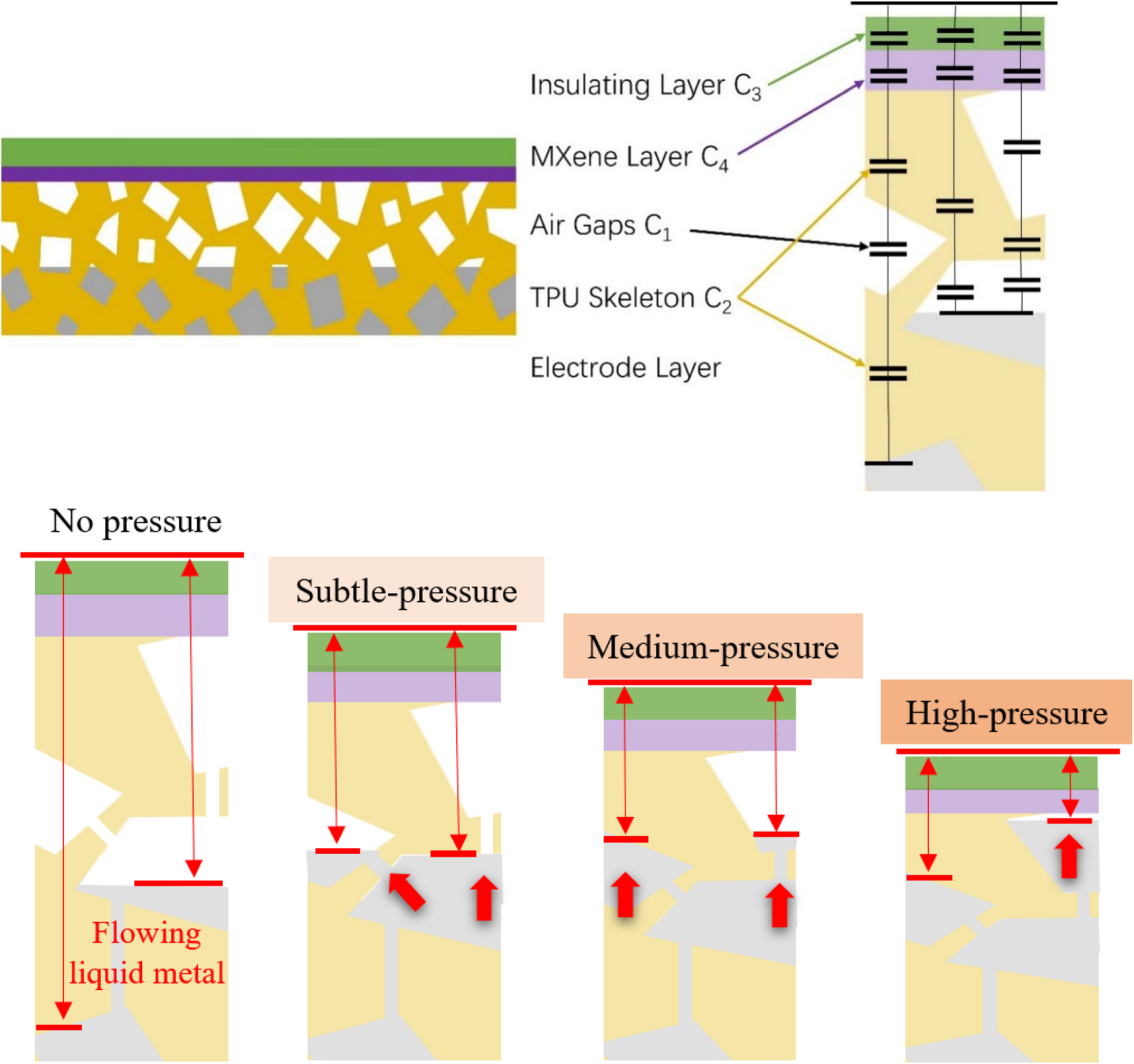
Schematic illustration of the capacitive sensing mechanism for the as-proposed TMLS.

For the 3D dynamic flowing liquid metal embedded in the TPU porous interconnected foam skeleton, the formation of new oxides of liquid metal during compression is effectively suppressed due to its high surface energy and low surface area to volume ratio, which can effectively prevent the denaturation of liquid metal and enhance its durability. In addition, when the external pressure applied to the flexible capacitive pressure sensor causes the TPU skeleton to be compressed, the liquid metal flows dynamically into the interconnected pores of the TPU foam and always maintains a path network of continuous current conduction, which makes the conductivity of the electrode layer remains stable even under a considerable load change cycle. These aspects ensure that TMLS exhibits a stabilized output response across a broad pressure range, thereby ensuring its robust and reliable performance in practical applications over an extended period (Fig. 5).

### 3.4 Demonstrations for TMLS

For the purpose of further proving the above excellent sensing performance and other related advantages of TMLS, we designed several experiments to verify its application in the field of flexible wearable devices.

**Fig. 6:**
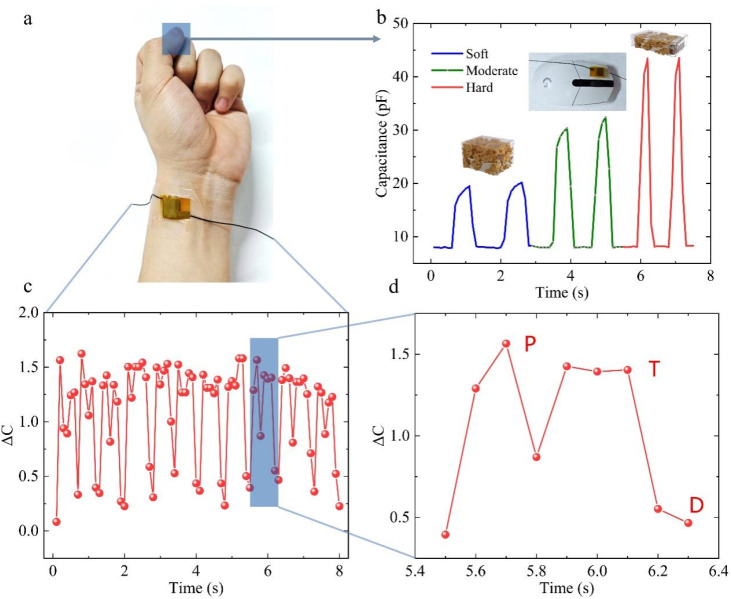
The performance of our proposed capacitive sensor as a wearable electronic. (a) Photograph of TMLS installed on the skin over the wrist radial artery. (b) Capacitive response triggered by finger pressing the mouse with different pressures. (c) Capacitive response of the radial artery pulse detected by TMLS in real time, and (d) a local enlarged view showing the characteristic peaks.

In the field of human machine integration, we attached TMLS to the left mouse button. It can be seen from Fig. 6b that TMLS can have a good and fast capacitance response to both slight pressure and large pressure by clicking on the mouse with fingers under different pressures. With the help of TMLS, the signal output of the mouse's original press/release was transformed into a signal output that can respond to pressure in real time.

Recent research has consistently focused on electronic skin. The pivotal aspect of electronic skin lies in its ability to achieve pressure monitoring, enabling the quantification of motion data. Here we demonstrate the sensor is fixed with the volunteer's wrist above the radial artery (Fig. 6a). In order to clearly record the arterial pulse wave without slipping off, as shown in the figure, medical tape was employed for wrapping. The real-time capacitance signal output curve (Fig. 6c) demonstrates that when the TMLS is connected to the wrist, it can produce a clear capacitance response signal to the movement of the blood vessel. It can also be analyzed that the output frequency of the sitting pulse signal is about 82 times per minute. Three regions can be observed and distinguished in the enlarged image of Fig. 6d, which correspond to the shock wave, tidal wave, and diastolic wave of the pulse signal respectively. These are important indicators for assessing human health, indicating that TMLS has the sensitivity and response time suitable for detecting radial artery blood pressure waves and distinguishing their related peaks. In general, all of these test results underscore the hope that our proposed sensors have further applications in the creation of electronic skin with similar human sensory abilities.

## 4. Conclusions

In summary, we report an easy-to-fabricate and high-performance flexible capacitive sensor based on interconnected-porous TPU/MXene composite with embedded liquid-metal (LM). Our composite structure design includes the preparation of electrode layers of interconnected-porous TPU embedded with liquid-metal (LM) and ultrathin MXene self-supported thin-film dielectric layers. The interconnecting porous TPUs were prepared by the sacrificial template method and modified to be embedded with LM, and the MXene self-supported films were prepared by vacuum-assisted filtration. Experiments and data show that, unlike conventional conductive unit-filled composites, the metallic conductivity and fluid ductility of the embedded LM effectively enhance the pressure sensitivity of the capacitive sensors, while the incompressible property of the embedded fluid LM results in the pores of the composite foam not to be easily collapsed when subjected to high-pressure loads, which results in a wider pressure sensing range of our sensors. The sensor has a sensitivity as high as 1.91 kPa^−1^, a wide detection range of 10 Pa–260 kPa, and excellent reliability over 4000 long-cycle loading cycles, with response times of only 60 ms and 110 ms during loading and unloading, respectively. In addition, our sensor is easy to construct by the sacrificial template method, and the preparation process is simple, low-cost, and easy to realize large-scale repetitive preparation. In addition, our sensors are easy to construct by the sacrificial template method, simple and low-cost preparation process, and easy to realize large-scale repetitive preparation. With these excellent characteristics, TMLS can be used for multi-range response recognition of pressed fingers in proof-of-concept synthesis. In addition, the good sensing characteristics also provide the basic conditions for detecting the tiny beats of the radial artery pulse wave, completing the identification and successfully demonstrating the good application prospects in motion detection and monitor biophysical heart-rate signals. Finally, we believe that this design mechanism based on 3D liquid metal electrodes can be easily generalized to the structural design of other sensors as a strategic means of optimizing performance of the next-generation wearable electronics.

## Author contributions

Jing Li and Zhong Zheng: original idea and design of methodology. Xing Fang: carry out the synthesis of materials. Zhong Zheng and Xing Fang: performing the experiments and data collection. Jun Li, Yifan Pan, and Shuyi Song participated in the discussion of the experimental plan and assisted in the experiment. Zhong Zheng and Yi Li: writing the initial draft. Huan Xue and Zhong Zheng: supervision, resources and funding acquisition. All the authors participated in writing - review and editing.

## Conflicts of interest

The authors declare that they have no known competing financial interests or personal relationships that could have appeared to influence the work reported in this paper.

## Supplementary Material

RA-014-D4RA01215A-s001
