# Supplementary material for: A liquid metal-embedded 3D interconnected-porous TPU/MXene composite with improved capacitive sensitivity and pressure detection range
Source: RSC Adv. 2024 May 14;14(22):15730–41. doi: 10.1039/d4ra01215a (PMC11092364; doi:10.1039/d4ra01215a)
Supplement: RA-014-D4RA01215A-s001 [file RA-014-D4RA01215A-s001.pdf]

Electronic Supplementary Material (ESI) for RSC Advances.
This journal is © The Royal Society of Chemistry 2024

# Supporting Information

## Liquid metal-embedded 3D interconnected-porous TPU\MXene composite with improved capacitive sensitivity and pressure detection range

*Zhong Zheng[a], Xing Fang[a], Yifan Pan[a], Shuyi Song[a], Huan Xue[a], Jun Li[a], Yi Li[b], Jing Li[a,*]*

[a] Hubei Key Laboratory of Modern Manufacturing Quantity Engineering, School of Mechanical Engineering, Hubei University of Technology, Wuhan, Hubei, 430068, China.

[b] Renal Division and Division of Engineering in Medicine, Department of Medicine, Brigham and Women's Hospital, Harvard Medical School, Boston, MA 02115, USA.

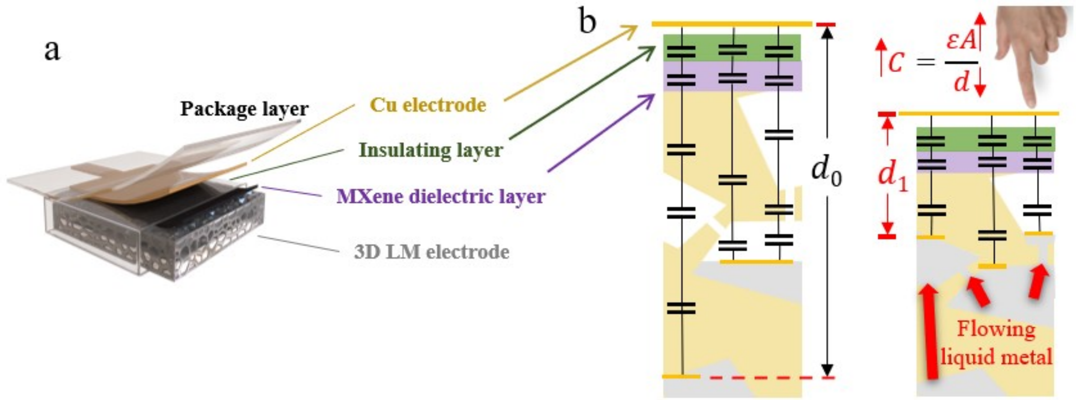


**Fig S1. Schematic of construction of the as-proposed TMLS.** (a) Exploded schematic illustration of a TMLS. Schematic illustration of (b) mechanical deformation behavior of TMLS during pressure loading.

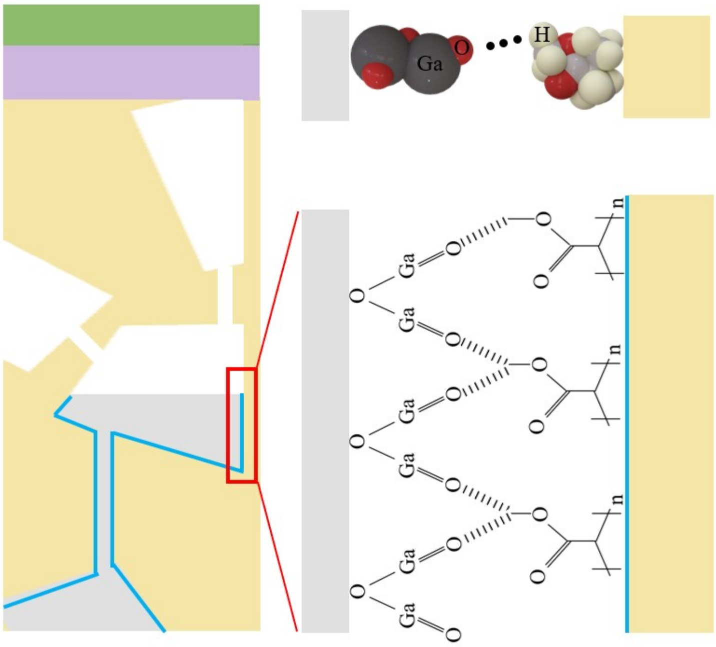


**Fig S2. The schematic diagrams of the chemical interaction between PMA and EGaIn.**

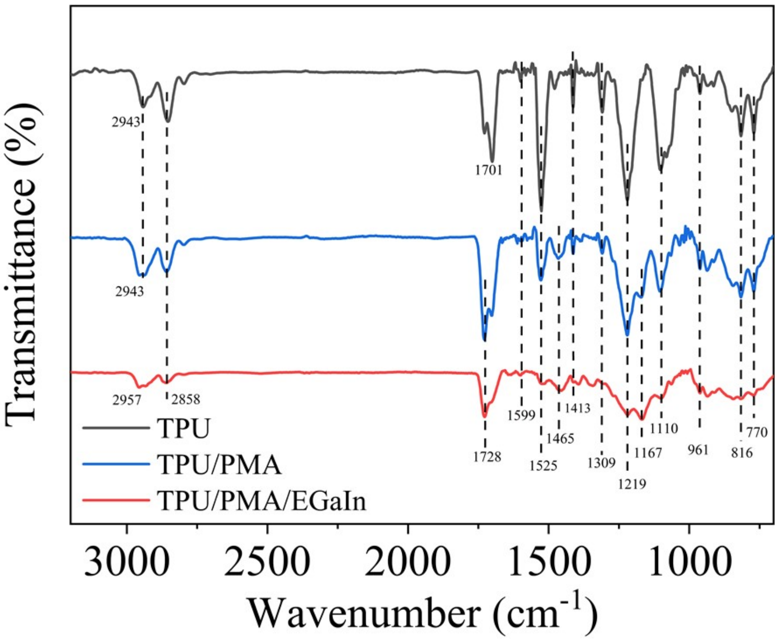


**Fig S3. FT-IR spectra of TPU, TPU/PMA composite foam, and TPU/PMA/EGaIn composite foam.**

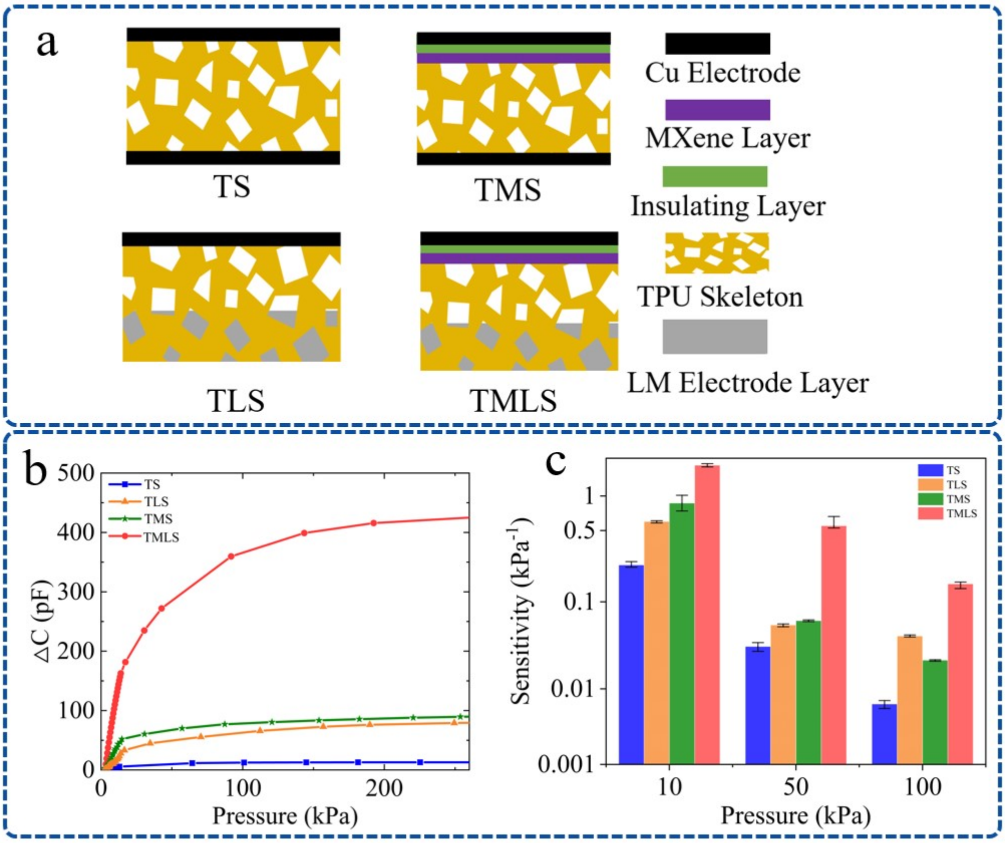


**Fig S4. Comparison of TMLS and other capacitive pressure sensor counterparts.** (a) Schematic illustration of the three conventional capacitive pressure sensors and TMLS (b) Pressure-capacitance variation curve of the four sensors. (c) Sensitivity comparison of the four sensors.

**Table S1** A comparison of the sensing performance of reported sensors[1-19].

| Ref. | Maximum Sensitivity ($kPa^{-1}$) | Maximum detectable pressure (kPa) | Minimum Detection limit | Response/relaxation time (ms) | Durability (cycles) |
|---|---|---|---|---|---|
| This work | 1.91 | 260 | 10 Pa | 60/110 | 4000 |
| [1] | 0.49 | 11 | 20 Pa | 30/30 | 300 |
| [2] | 1.21 | 28 | 0.9 Pa | 100/100 | 10000 |
| [3] | 0.283 | 33 | - | - | 20000 |
| [4] | 0.141 | 100 | 0.16 g | 190/210 | 1000 |
| [5] | 0.065 | 1700 | - | 100/100 | 7000 |
| [6] | 0.201 | 4500 | - | 60/30 | 1000 |
| [7] | 6.583 | 1 | 3 Pa | 48/36 | 10000 |
| [8] | 0.37 | 115 | - | 84/105 | 7600 |
| [9] | 4.4 | 115 | 0.8 Pa | 16/46 | 50000 |
| [10] | 0.854 | 2.1 | 1 Pa | - | - |

| | | | | | |
|---|---|---|---|---|---|
| [11] | 2.1 | 70 | 1.3 Pa | 50/50 | 3600 |
| [12] | 0.41 | 55 | 0.005g | 33/33 | 1000 |
| [13] | 0.18 | 400 | 10 Pa | 100/100 | 10000 |
| [14] | 0.159 | 145 | - | 49/51 | 9000 |
| [15] | 4.2 | 32 | 1.6 Pa | 26/26 | 10000 |
| [16] | 4.05 | 100 | 0.6 Pa | 26/52 | 14000 |
| [17] | 0.023 | 200 | - | 155/- | 1000 |
| [18] | 0.124 | 150 | - | 167/117 | 1000 |
| [19] | 0.055 | 10 | - | 200/150 | - |

## Reference


1. Z. H. Wang, Y. Si, C. Y. Zhao, D. Yu, W. Wang and G. Sun, *Acs Applied Materials & Interfaces*, 2019, **11**, 27200-27209.
2. S. F. Zhao, W. H. Ran, D. P. Wang, R. Y. Yin, Y. X. Yan, K. Jiang, Z. Lou and G. Z. Shen, *Acs Applied Materials & Interfaces*, 2020, **12**, 32023-32030.
3. R. H. Wu, L. Y. Ma, A. Patil, C. Hou, S. H. Zhu, X. W. Fan, H. Z. Lin, W. D. Yu, W. X. Guo and X. Y. Liu, *Acs Applied Materials & Interfaces*, 2019, **11**, 33336-33346.
4. S. Lee, S. Franklin, F. A. Hassani, T. Yokota, O. G. Nayeem, Y. Wang, R. Leib, G. Cheng, D. W. Franklin and T. Someya, *Science*, 2020, **370**, 966-+.
5. B. Ji, Q. Zhou, M. Lei, S. Ding, Q. Song, Y. Gao, S. Li, Y. Xu, Y. Zhou and B. Zhou, *Small*, 2021, **17**.
6. T. Y. Shao, J. N. Wu, Y. H. Zhang, Y. R. Cheng, Z. Q. Zuo, H. K. Lv, M. L. Ying, C. P. Wong and Z. Li, *Advanced Materials Technologies*, 2020, **5**.
7. H. S. Niu, S. Gao, W. J. Yue, Y. Li, W. J. Zhou and H. Liu, *Small*, 2020, **16**.
8. A. Chhetry, S. Sharma, H. Yoon, S. Ko and J. Y. Park, *Advanced Functional Materials*, 2020, **30**.
9. M. Fu, J. M. Zhang, Y. M. Jin, Y. Zhao, S. Y. Huang and C. F. Guo, *Advanced Science*, 2020, **7**.
10. R. Y. Tay, H. L. Li, J. J. Lin, H. Wang, J. S. K. Lim, S. Chen, W. L. Leong, S. H. Tsang and E. H. T. Teo, *Advanced Functional Materials*, 2020, **30**.
11. L. X. Mo, X. Y. Meng, J. Zhao, Y. Q. Pan, Z. C. Sun, Z. X. Guo, W. Wang, Z. C. Peng, C. Shang, S. B. Han, K. Hu, M. J. Cao, Y. J. Chen, Z. Q. Xin, J. S. Lu and L. H. Li, *Flexible and Printed Electronics*, 2021, **6**.
12. Z. B. Luo, J. Chen, Z. F. Zhu, L. Li, Y. Su, W. Tang, O. M. Omisore, L. Wang and H. Li, *Acs Applied Materials & Interfaces*, 2021, **13**, 7635-7649.
13. J. Hwang, Y. Kim, H. Yang and J. H. Oh, *Composites Part B-Engineering*, 2021, **211**, 9.
14. W. Asghar, F. L. Li, Y. L. Zhou, Y. Z. Wu, Z. Yu, S. B. Li, D. X. Tang, X. T. Han, J. Shang, Y. W. Liu and R. W. Li, *Advanced Materials Technologies*, 2020, **5**.
15. W. Yang, N. W. Li, S. Y. Zhao, Z. Q. Yuan, J. N. Wang, X. Y. Du, B. Wang, R. Cao, X. Y. Li, W. H. Xu, Z. L. Wang and C. J. Li, *Advanced Materials Technologies*, 2018, **3**.
16. M. Su, J. T. Fu, Z. X. Liu, P. Li, G. J. Tai, P. S. Wang, L. Xie, X. Q. Liu, X. M. He, D. P. Wei and J. Yang, *Acs Applied Materials & Interfaces*, 2023, **15**, 48683-48694.

17. S. M. Li, K. Dong, R. Q. Li, X. Y. Huang, T. J. Chen and X. L. Xiao, *Sensors and Actuators a-Physical*, 2020, **312**, 9.
18. Bijender and A. Kumar, *Acs Omega*, 2020, **5**, 16944-16950.
19. C. Mahata, H. Algadi, J. Lee, S. Kim and T. Lee, *Measurement*, 2020, **151**.
